# Supplementary material for: Splice-Junction-Based Mapping of Alternative Isoforms in the Human Proteome
Source: Cell Rep. Author manuscript; Available in PMC 2020 Jan 15. (PMC6961840; doi:10.1016/j.celrep.2019.11.026)
Supplement: 2 [file NIHMS1546469-supplement-2.zip › DF1/PXD010154/Spleen/Spleen_8_TNXB_MGPLSVVIVTVAR.pdf]

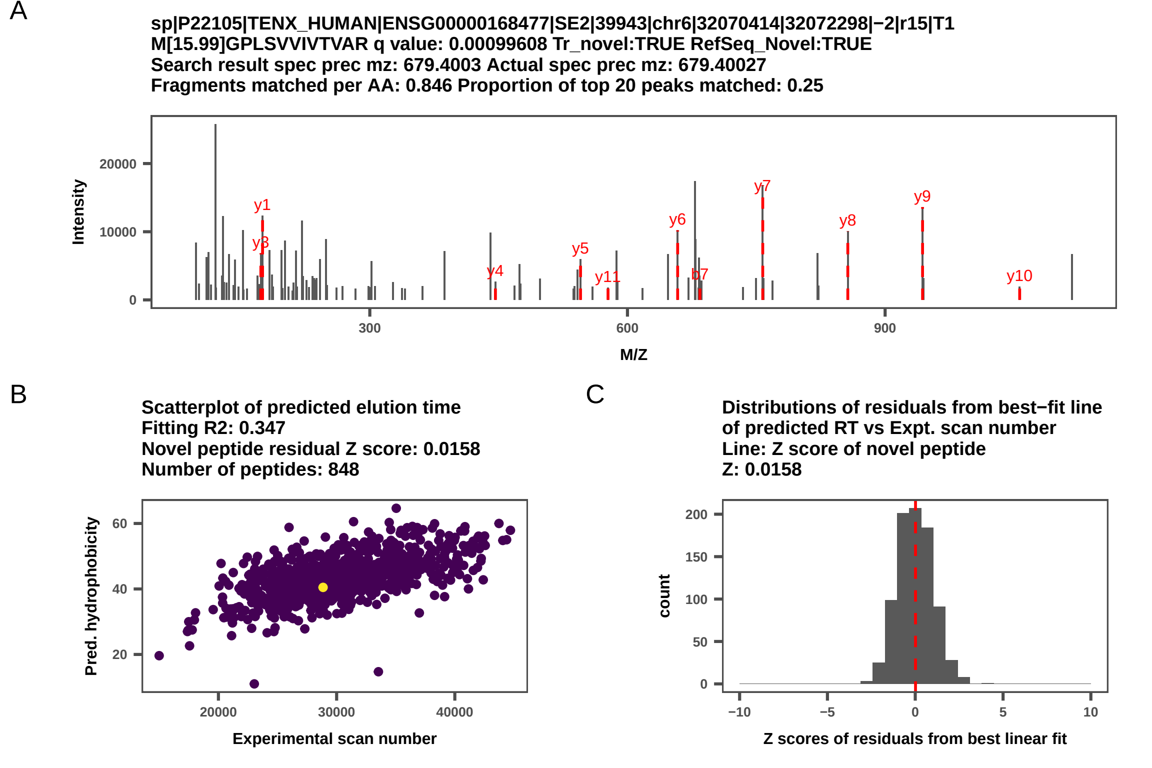
A
sp|P22105|TENX_HUMAN|ENSG00000168477|SE2|39943|chr6|32070414|32072298|−2|r15|T1
M[15.99]GPLSVVIVTVAR q value: 0.00099608 Tr_novel:TRUE RefSeq_Novel:TRUE
Search result spec prec mz: 679.4003 Actual spec prec mz: 679.40027
Fragments matched per AA: 0.846 Proportion of top 20 peaks matched: 0.25
Intensity
20000
10000
0
y1
y3
y4
y5
y11
y6
b7
y7
y8
y9
y10
300
600
900
M/Z
B
Scatterplot of predicted elution time
Fitting R2: 0.347
Novel peptide residual Z score: 0.0158
Number of peptides: 848
Pred. hydrophobicity
60
40
20
20000
30000
40000
Experimental scan number
C
Distributions of residuals from best−fit line
of predicted RT vs Expt. scan number
Line: Z score of novel peptide
Z: 0.0158
count
200
150
100
50
0
−10
−5
0
5
10
Z scores of residuals from best linear fit
